# Supplementary material for: A Mutation in DNA Polymerase α Rescues WEE1KO Sensitivity to HU
Source: Int J Mol Sci. 2021 Aug 30;22(17):9409. doi: 10.3390/ijms22179409 (PMC8430855; doi:10.3390/ijms22179409)
Supplement: Supplementary file 1 [file ijms-22-09409-s001.zip › ijms-1312551-supplementary.pdf]

Supplementary Materials:

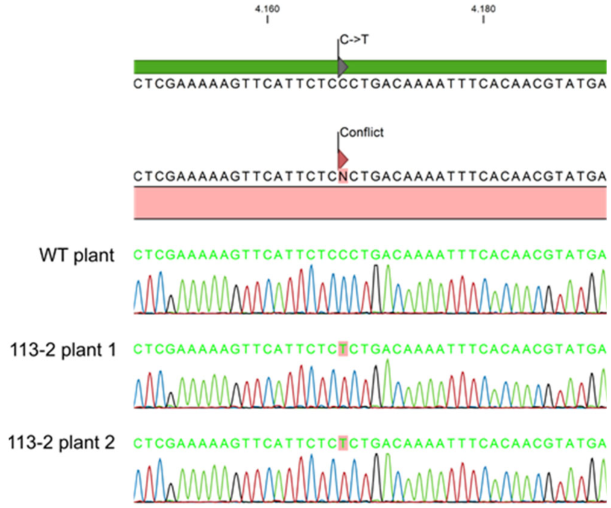

Figure S1. Confirmation of mutation in line 113-2 by Sanger sequencing.

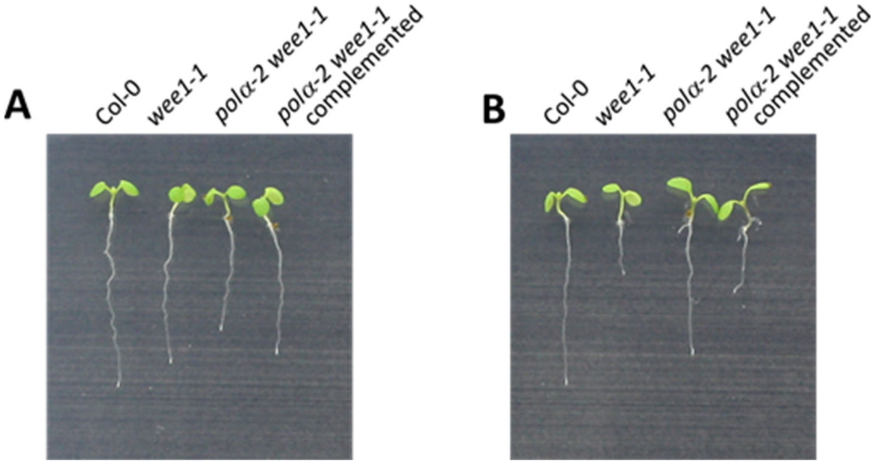

Figure S2. A complementation construct restores HU-sensitivity to *polα-2 wee1-1* plants. Root growth of 7-day-old wild-type (Col-0), *wee1-1* and *polα-2 wee1-1* seedlings, together with *polα-2 wee1-1* seedlings transformed with the complementation construct, grown on control medium (A) or medium supplemented with 0.75 mM HU (B).

Supplemental table S1

wee1-1_genotype_FW	TCAATAAGGCTTGGTTTCTTCAGT
wee1-1_genotype_REV	CCCTACGAACAAAACAAGATAAAAAC
wee1-1_TDNA_REV	ATAATAACGCTGCGGACATCTACATTTT
atm-2_genotype_FW	GGTTGGGCAGTTCCAAAGATGA
atm-2_genotype_REV	TCTCTCCTTGTTTCAAGCTCTG
atr-2_genotype_FW	CAAGGGTTCCGATGTTCAAAGTG
atr-2_genotype_REV	CAATCAGCAGGAAAAGACAAATC
SALK_TDNA_FW	ATTTTGCCGATTTCGGAAC
sog1-1_genotype_FW	CGATCATGGCTGATCGATAG
sog1-1_genotype_FW	TCGGCAAGAGAAACCTCATG
pola-2_genotype_FW	TCACTCTCCAGCTTACTAACA
pola-2_genotype_REV	GTAGGCTGGACCTTTTTTTGT
EMB2386_qRT_FW	CTCTCGTTCCAGAGCTCGCAAAA

EMB2386_qRT_REV	AAGAACACGCATCCTACGCATCC
PAC1_qRT_FW	TCTCTTTGCAGGATGGGACAAGC
PAC1_qRT_REV	AGACTGAGCCGCCTGATTGTTTG
RPS26C_qRT_FW	GACTTTCAAGCGCAGGAATGGTG
RPS26C_qRT_REV	CCTTGTCCTTGGGGCAACACTTT
BRCA1_qRT_FW	TGTTCCCTCTTTCAGCGATTTGATG
BRCA1_qRT_REV	GGCCTCTGAGTCCATTCAAACA
PARP2_qRT_FW	ATGGCGTTCTGCTCCTCTGC
PARP2_qRT_REV	GGTGCTGTTTTCCCCACACC
RAD17_qRT_FW	TGTGCACAACACTAGTTCAGGAC
RAD17_qRT_REV	AACAACGGGCGCCTTTATTCC
RAD51_qRT_FW	TTCCGCTCTGGAAAGACTCAGC
RAD51_qRT_REV	ACCTCCTTGATCCATGGGAAGTTG
SMR4_qRT_FW	GCCGAGAAGCACGATGTATAG
SMR4_qRT_REV	AGATCTGGTGGCTGAAAGTACC
SMR5_qRT_FW	AAACTACGACGACGGAGATACG
SMR5_qRT_REV	GCTACCACCGAGAAGAACAAGT
SMR7_qRT_FW	TTCACTAAAGCCGGTGAAGACG
SMR7_qRT_REV	CGCCGTGGGAGTGATACAAATTC
TSO2_qRT_FW	TGTCCGATGTTCAAGTCTCTGAG
TSO2_qRT_REV	CTCCGAGTGGATGTTTTCAATCG
AP3_qRT_FW	CCCTAACACCACAACGAAGGAGAT
AP3_qRT_REV	GTTTCCTCTTGGTTTCTTGCATTC
SEP3_qRT_FW	TTAGCAGTTGAACTTAGTAGCCA
SEP3_qRT_REV	CCAAGATCTTCTCCCAACAGAT
FT_qRT_FW	GAACAACCTTTGGCAATGAGATT
FT_qRT_REV	CACCCTGGTGCATACACTGTT
